# LINC01121 Is Associated With Prognosis and Facilitates the Proliferation, Migration, and Invasion of Colorectal Cancer

**DOI:** 10.14740/wjon2629

**Published:** 2025-12-17

**Authors:** Jiang Liu, Ying Xuan Zhang, Yin Cai, Xiang Wu, Jian He Yu, Da Dong Chen, Wei Yu Chen, Chuan Jun Song

**Affiliations:** a Department of Oncology, The Affiliated Xinghua People’s Hospital, Medical School of Yangzhou University, Xinghua, Jiangsu 225700, China; b Department of Integrative Oncology, Fudan University Shanghai Cancer Center Xiamen Hospital, Xiamen, China; c These authors contributed equally to this work.

**Keywords:** LINC01121, Colorectal cancer, Biomarkers, Prognosis

## Abstract

**Background:**

The role of LINC01121 in the pathogenesis and prognosis of colorectal cancer (CRC) remains unknown. This study aimed to explore the function of LINC01121 in CRC development.

**Methods:**

Transcriptome expression data from The Cancer Genome Atlas (TCGA) database were utilized to investigate the relationship between LINC01121 and CRC prognosis through survival analysis. Samples from CRC patients and adjacent tissues were collected and analyzed to assess the expression differences between tumor and adjacent tissues. Functional assays such as Cell Counting Kit-8 (CCK8), EdU, scratch test, and Transwell assay were employed to determine the oncogenic role of LINC01121 in CRC cells. Additionally, gene enrichment analyses and immune infiltration analyses were conducted with R package.

**Results:**

The LINC01121 level was obviously higher in CRC tissues. The receiver operating characteristic (ROC) curve suggests that LINC01121 has significant diagnostic capabilities (area under the curve (AUC) = 0.659). High expression of LINC01121 predicted poor overall survival (OS) (P = 0.022), progression-free interval (PFI) (P = 0.007), and disease-specific survival (DSS) (P = 0.006). Gene Set Enrichment Analysis (GSEA) demonstrated that LINC01121-associated CRC encompasses various crucial pathways linked to tumorigenesis. The immune infiltration analyses revealed that LINC01121 may be involved in immune suppression. *In vitro* experiments demonstrated that LINC01121 facilitated the proliferation, migration, and invasion of CRC cells.

**Conclusion:**

LINC01121 exhibits elevated expression in CRC and correlates with unfavorable prognosis and reduced immune infiltration in CRC patients. These findings suggest that LINC01121 may serve as a potential marker for the diagnosis and prognosis of CRC.

## Introduction

Colorectal cancer (CRC) is the main identifiable cause of cancer-related deaths globally. It is estimated that by 2040, the incidence rate of CRC in the world will increase to 3.2 million [1]. The primary causes of mortality in CRC are attributed to tumor progression-induced recurrence and metastasis [2, 3]. While the onset of CRC is associated with diet, microorganisms, and their metabolites, the precise mechanism remains unclear. Thus, identifying dependable molecular markers and comprehending the underlying mechanisms are vital to enhancing diagnosis and therapy of CRC.

Long non-coding RNA (lncRNA) is a class of RNA molecules with transcript lengths exceeding 200 nt, involved in the regulation of protein coding genes at different levels such as epigenetics, transcription, and post-transcriptional regulation [4]. LncRNAs are broadly implicated in various diseases, including tumors, due to their regulatory functions [5, 6]. Nevertheless, numerous lncRNA expressions are aberrantly regulated in CRC and participate in its occurrence and development [7, 8]. The specific molecular mechanism of LINC01121 in cancer is still unclear. It has been reported that LINC01121 promoted the development of breast cancer cells through the miR-150-5p/HMGA2 axis [9]. In another study, LINC01121 may be involved in the process of pterostilbene inhibiting breast cancer cell proliferation and epithelial mesenchymal transformation (EMT) [10]. LINC01121 has not been previously examined in CRC. Our research highlights elevated levels of LINC01121 in CRC, correlating with unfavorable patient outcomes. Functional analyses indicate that LINC01121 is instrumental in CRC progression, facilitating both growth and metastasis. As a result, this study elucidates the potential mechanisms and clinical significance of LINC01121 in CRC, offering new perspectives on the disease’s pathogenesis.

## Materials and Methods

### Data source

We gathered gene expression data (HTSeq-TPM) and related information for a sum of 644 CRC cases from COAD-ROAD project of The Cancer Genome Atlas (TCGA), and those without clinic information were rejected. We eliminated normal COAD-ROAD samples and those with an overall survival (OS) rate of less than 30 days as exclusion criteria.

### Clinical statistical analyses

In this study, statistical analyses were conducted using the R package (version 4.2.2). The median expression level of LINC01121 served as the cutoff for categorizing samples into two groups: high and low expression. A P-value of less than 0.05 was deemed statistically significant across all analyses. The diagnostic performance of LINC01121 expression was analyzed via the pROC package, which produced a receiver operating characteristic (ROC) curve. Nomograms were developed based on the multivariate analysis results to customize the estimated 1-, 3-, and 5-year survival probabilities. The RMS R package was used to plot bar charts that incorporated clinically relevant features and calibration plots associated with LINC01121.

### GSEA

In this research, the R package ClusterProfiler [11] was utilized to conduct Gene Set Enrichment Analysis (GSEA), a computational approach that assesses whether there are any significant consistent differences between two biological conditions in a specified gene set, such as high-LINC01121 and low-LINC01121 groups. The analysis process was replicated 1,000 times and an adjusted P-value < 0.05 and false discovery rate (FDR) < 0.25 was used to determine statistically meaningful enrichment for a function or pathway term.

### Immune infiltration analysis by ssGSEA

Utilizing the ssGSEA method from the GSVA R package (version 3.6), we quantified the infiltration levels of 24 immune cell types based on gene expression data from previous studies [12] and investigated the association between LINC01121 and these immune cell infiltration levels.

### Tissues and cells

Thirty pairs of cancer and adjacent samples of human CRC patients who underwent radical surgery at Xinghua People’s Hospital from January 2023 to September 2023 were collected. The human CRC cell line was obtained from the Chinese Academy of Sciences (Shanghai, China).

### RT-PCR

Total cell RNA was extracted using TRIzol reagent (Invitrogen, USA), followed by PCR amplification of all transcripts with the 2X SYBR Green qPCR Master Mix kit (Abm, Canada). The primer sequence utilized is given below: LINC01121 (F: AACAGGCAATAAGGCAAGAATCGC, R: TCCAGCAGGCTCAGAAGGCACA). Use β-actin as standard.

### Cell transfection

The siRNA sequences (si-1: TTCAACTGACAACAGTAAATA, si-2: TGGGTCAAATGACACAATTAA) and siRNA-NC were obtained from RiboBio and transfected with Lipofectamine 2000 (Invitrogen, USA).

### Cell proliferation assay

For cell survival assays in 96-well plates, the Cell Counting Kit-8 (CCK8) from Beyotime (China) was used according to the protocol.

### Scratch healing assay

To conduct cell migration experiments, cells were grown in six-well culture plates. Scratch wounds were created in the cell monolayer using pipette tips. Over time, cell movement towards the wound surface was observed at 48-h intervals.

### Transwell assays

For transwell invasion assays, Transwell chambers (BD Biosciences, USA) with Matrigel were used. Serum-starved cells (5 × 10^4^) were seeded in the upper wells using serum-free medium. The lower chamber was filled with 600 µL of complete medium. After a period of 48 h, the cells that had invaded through the matrix were fixed using 4% paraformaldehyde, stained using 0.1% crystal violet, and counted under a microscope.

### Western blot analysis

Cells were lysed using RIPA lysis buffer (Merck, China), and protein concentration was standardized with the BCA method. The lysates were separated on 10-15% SDS-polyacrylamide gels and transferred to PVDF membranes. Subsequently, the membranes were incubated overnight at 4 °C with primary antibodies: anti-E-cadherin (1:1,000, CST, USA), anti-N-cadherin (1:1,000, CST, USA), and anti-GAPDH (1:5,000, CST, USA). This was followed by a 2-h incubation at 25 °C with appropriate secondary antibodies (anti-rabbit, Immunoway, USA). Finally, the blots were developed using ECL.

### Statistical analyses

R (V 4.2.2) was employed for all statistical analyses in this study. The ggplot2 R package was utilized to depict the differences in expression.

### Ethical statements

This study complies with the Helsinki Declaration and has been approved by Medical Ethics Committee of the Xinghua People’s Hospital (JSXHRYL-NK-202305).

## Results

### LINC01121 is upregulated in CRC

**Figure 1:**
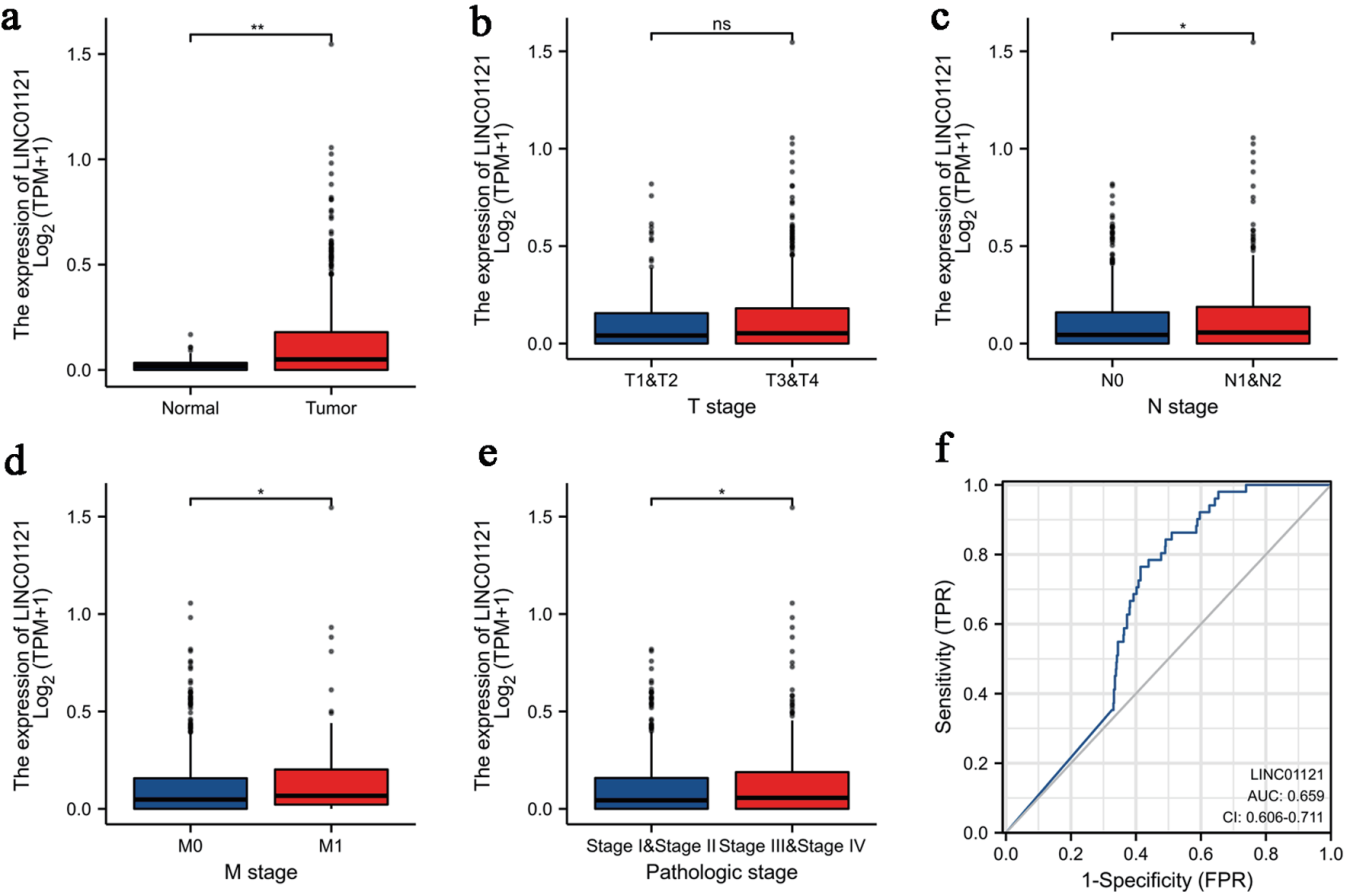
Association of LINC01121 expression with clinicopathologic characteristics: (a) expression level of LINC01121 in CRC samples and normal tissues; (b) LINC01121 expression in T1-2 stage patients compared to T3-4 stage; (c) LINC01121 expression in lymphatic metastasis-positive patients compared to those without lymphatic metastasis; (d) LINC01121 expression in M0 stage compared to M1 stage; (e) expression of LINC01121 in TNM stage I-II compared to III-IV; (f) ROC curve of LINC01121. *P < 0.05, **P < 0.01, ***P < 0.001, ****P < 0.0001. CRC: colorectal cancer; ns: not significant; ROC: receiver operating characteristic.

The clinical information of CRC patients from COAD-ROAD project of TCGA was shown in Table 1. LINC01121 expression was significantly higher in CRC tissues compared to normal tissues (Fig. 1a). However, there was no statistically difference observed in LINC01121 level between patients with T1-2 and T3-4 stage of CRC (Fig. 1b). The expression of LINC01121 was elevated in CRC patients with lymphatic metastasis-positive compared to those without lymphatic metastasis (Fig. 1c). Additionally, patients with distant metastasis demonstrated significantly higher levels of LINC01121 compared to those without distant metastasis (Fig. 1d). Furthermore, the expression of LINC01121 was upregulated in patients with stage III-IV compared to those with stage I-II (Fig. 1e). ROC analysis was conducted to evaluate the predictive value of LINC01121, and the area under the curve (AUC) was calculated to be 0.659 (95% confidence interval (CI): 0.606 - 0.717) (Fig. 1f).

**Table 1:** Clinical Information of Colorectal Patients in TCGA

Characteristics	Low expression of LINC01121	High expression of LINC01121	P value
n	322	322	
Gender, n (%)			0.693
Male	169 (26.2%)	174 (27%)	
Female	153 (23.8%)	148 (23%)	
Age, n (%)			0.152
≤ 65	129 (20%)	147 (22.8%)	
> 65	193 (30%)	175 (27.2%)	
Pathologic T stage, n (%)			0.118
T1	15 (2.3%)	5 (0.8%)	
T2	56 (8.7%)	55 (8.6%)	
T3	217 (33.9%)	219 (34.2%)	
T4	33 (5.1%)	41 (6.4%)	
Pathologic N stage, n (%)			0.568
N0	190 (29.7%)	178 (27.8%)	
N1	75 (11.7%)	78 (12.2%)	
N2	55 (8.6%)	64 (10%)	
Pathologic M stage, n (%)			0.191
M0	244 (43.3%)	231 (41%)	
M1	39 (6.9%)	50 (8.9%)	
Pathologic stage, n (%)			0.545
Stage I	58 (9.3%)	53 (8.5%)	
Stage II	125 (20.1%)	113 (18.1%)	
Stage III	89 (14.3%)	95 (15.2%)	
Stage IV	40 (6.4%)	50 (8%)	

TCGA: The Cancer Genome Atlas.

### High LINC01121 level is associated with adverse outcomes in CRC

**Figure 2:**
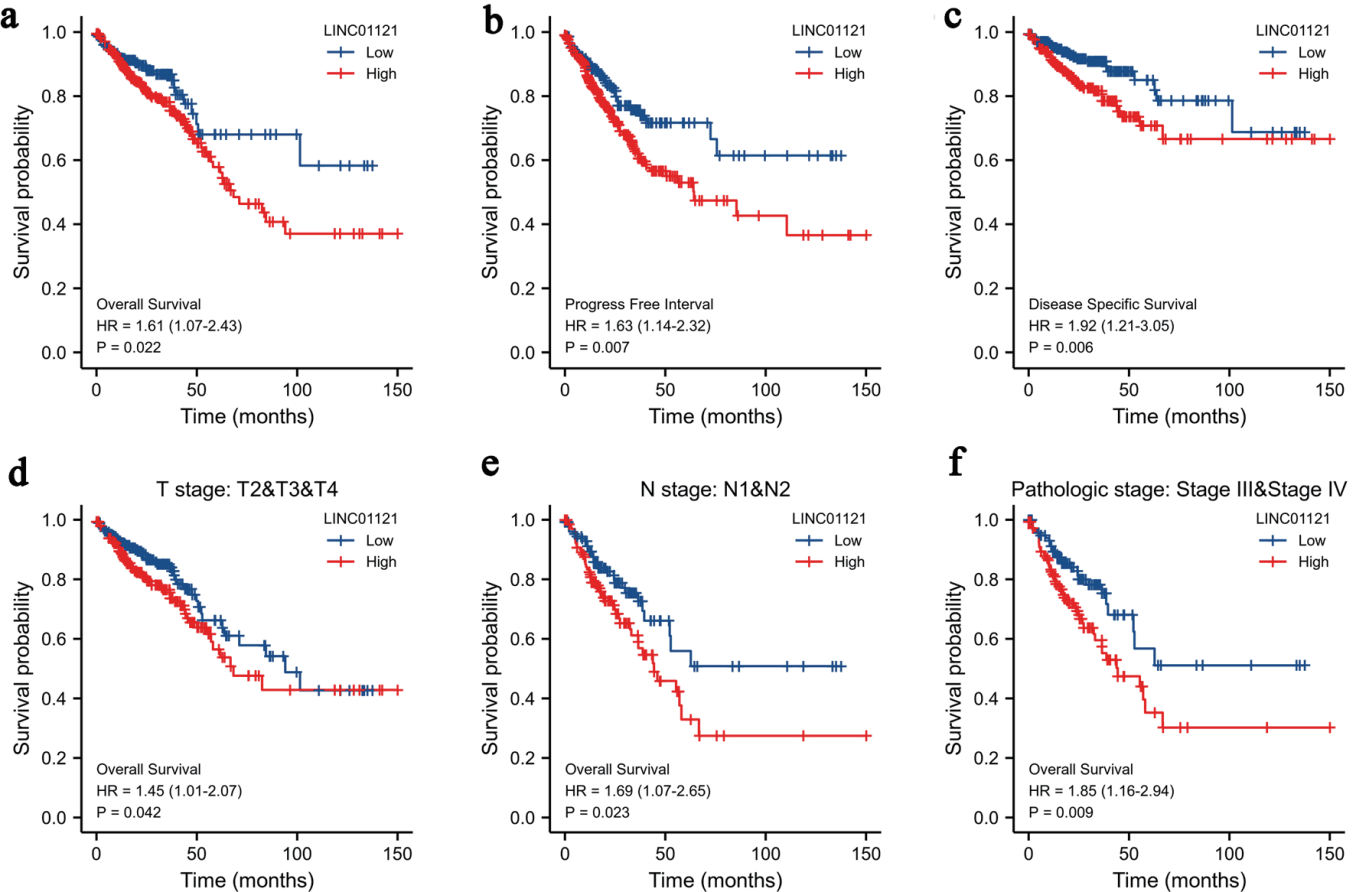
Kaplan-Meier survival curves comparing the high and low expression of LINC01121 in CRC patients: (a) overall survival; (b) progression-free interval; (c) disease-specific survival; (d-f) overall survival analyses of T2-T4, N1-2, and stages III and IV subgroup. CRC: colorectal cancer.

Elevated levels of LINC01121 were strongly correlated with a poor prognosis in patients diagnosed with CRC. Survival analysis results highlighted that compared to individuals with low expression of LINC01121, individuals with high LINC01121 expression experienced shorter OS, progression-free survival (PFS), and disease-specific survival (DSS) (Fig. 2a-c). Furthermore, when conducting stratified analyses, it was observed that in the samples classified as T2-4 group (Fig. 2d), lymph node metastasis group (Fig. 2e), and clinical stage III-IV group (Fig. 2f), patients exhibiting high levels of LINC01121 expression showed markedly poorer prognoses than those with lows LINC01121 level.

### Predictive value of LINC01121 for CRC prognosis

**Figure 3:**
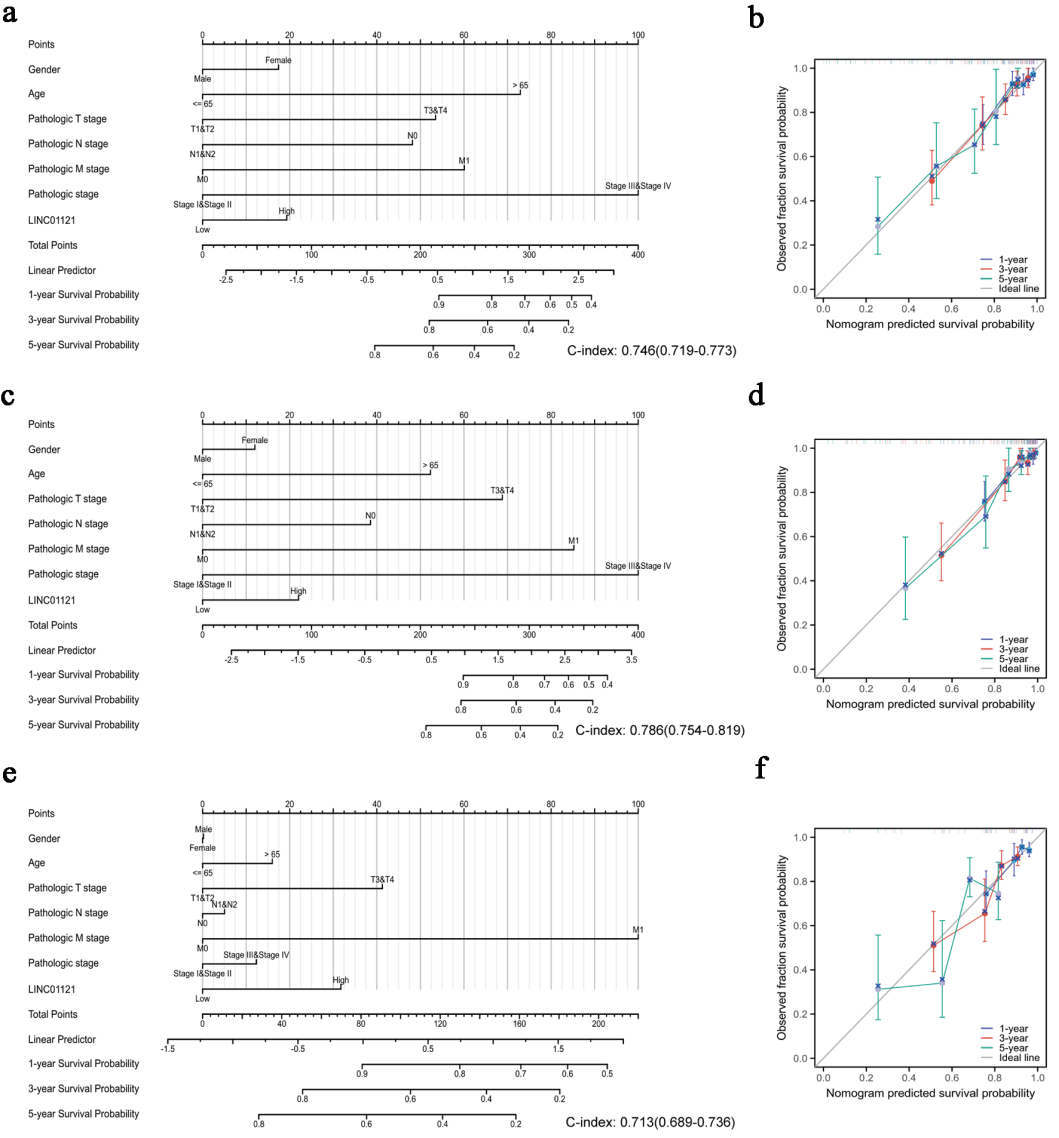
Quantitative methods to predict probability of 1-, 3-, 5-year OS, DSS, and PFI of CRC patients. Nomogram for predicting the probability of 1-, 3-, 5-year OS (a), DSS (c), and PFI (e) for CRC patients. Calibration plots of the nomogram for predicting the probability of OS (b), DSS (d), and PFI (f) at 1, 3, and 5 years. CRC: colorectal cancer; DSS: disease-specific survival; OS: overall survival; PFI: progression-free interval.

To establish a quantitative model to predict the prognosis of CRC patients, we constructed nomograms using LINC01121 and independent clinical risk factors for OS, DSS, and progression-free interval (PFI) (Fig. 3a, c, e). Utilizing multivariate Cox analysis, we assigned values to these factors in a point-based system: scores for each variable were determined along a straight line, and the cumulative score was subsequently adjusted to fall within the range of 0 to 100. Additionally, we created a calibration curve to evaluate the effectiveness of the nomogram. Our analysis revealed that the model’s C-index for OS was 0.746 (CI: 0.719 - 0.773), for DSS it was 0.786 (CI: 0.754 - 0.819), and for PFI it was 0.713 (CI: 0.689 - 0.736), indicating that the predictive accuracy of this model is moderately reliable. Furthermore, the calibration curve demonstrated strong predictions for clinical outcomes related to OS and DSS at 1, 3, and 5 years (Fig. 3b, d), although predictions for PFI were somewhat less precise (Fig. 3f). The results of univariate and multivariate regression analyses are presented in Supplementary Material 1 (wjon.elmerpub.com).

### Functional enrichment of LINC01121 in CRC

**Figure 4:**
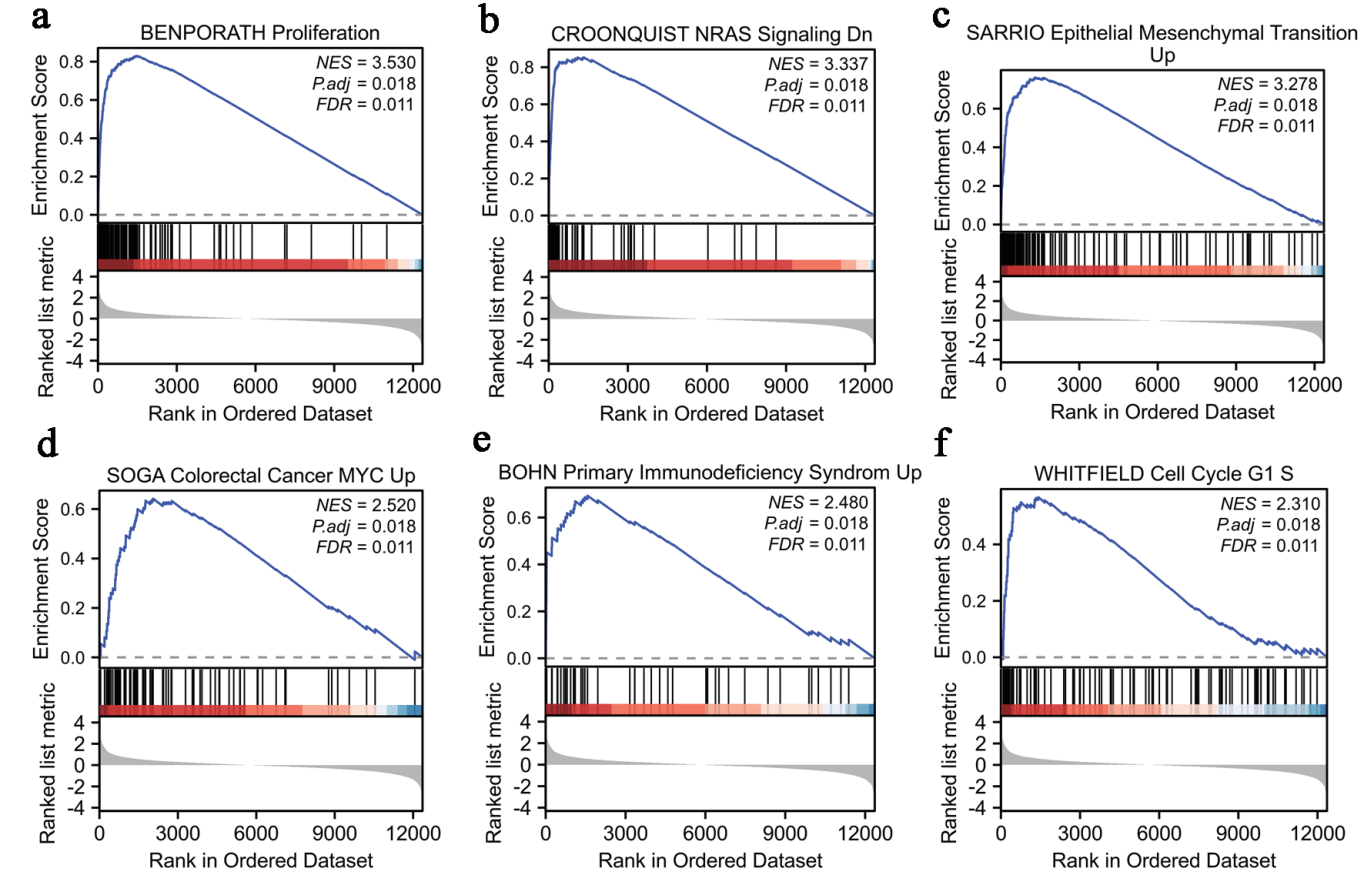
Enrichment analysis of LINC01121 in CRC: (a) proliferation; (b) NRAS signaling; (c) epithelial mesenchymal transition; (d) colorectal cancer MYC; (e) primary immunodeficiency syndrome; (f) cell cycle G1 S. CRC: colorectal cancer.

We utilized the normalized enrichment score (NES) to determine the most significant signaling pathways enriched in relation to LINC01121 expression. Figure 4 illustrates the results obtained from GSEA, demonstrating that LINC01121-associated CRC encompasses various crucial pathways linked to tumorigenesis. The results of enrichment pathways include proliferation, NRAS signaling pathway, epithelial mesenchymal transition, colorectal cancer MYC Up, primary immunodeficiency syndrome, and cell cycle (Fig. 4).

### LINC01121 is associated with immune suppression

**Figure 5:**
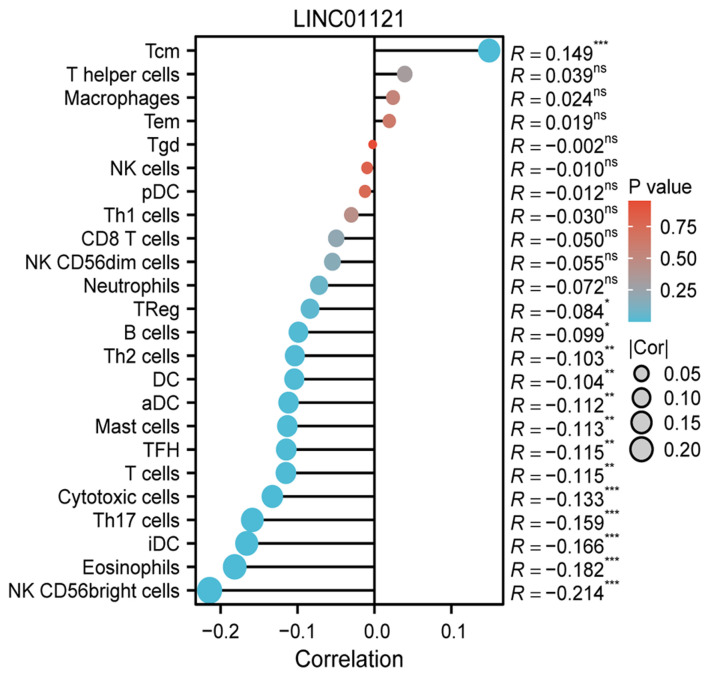
The relationship between LINC01121 expression and immune cell infiltration.

To investigate the relationship between LINC01121 expression and immune cell infiltration levels, we analyzed the correlation between immune cell infiltration and LINC01121 level. The results indicated that LINC01121 was negatively correlated with 13 types of immune cells, including natural killer (NK) CD56bright cells, Th17 cells, cytotoxic cells and eosinophiles, suggesting that LINC01121 may be involved in immune suppression (Fig. 5).

### LINC01121 mediates cell proliferation, migration, and invasion *in vitro*

**Figure 6:**
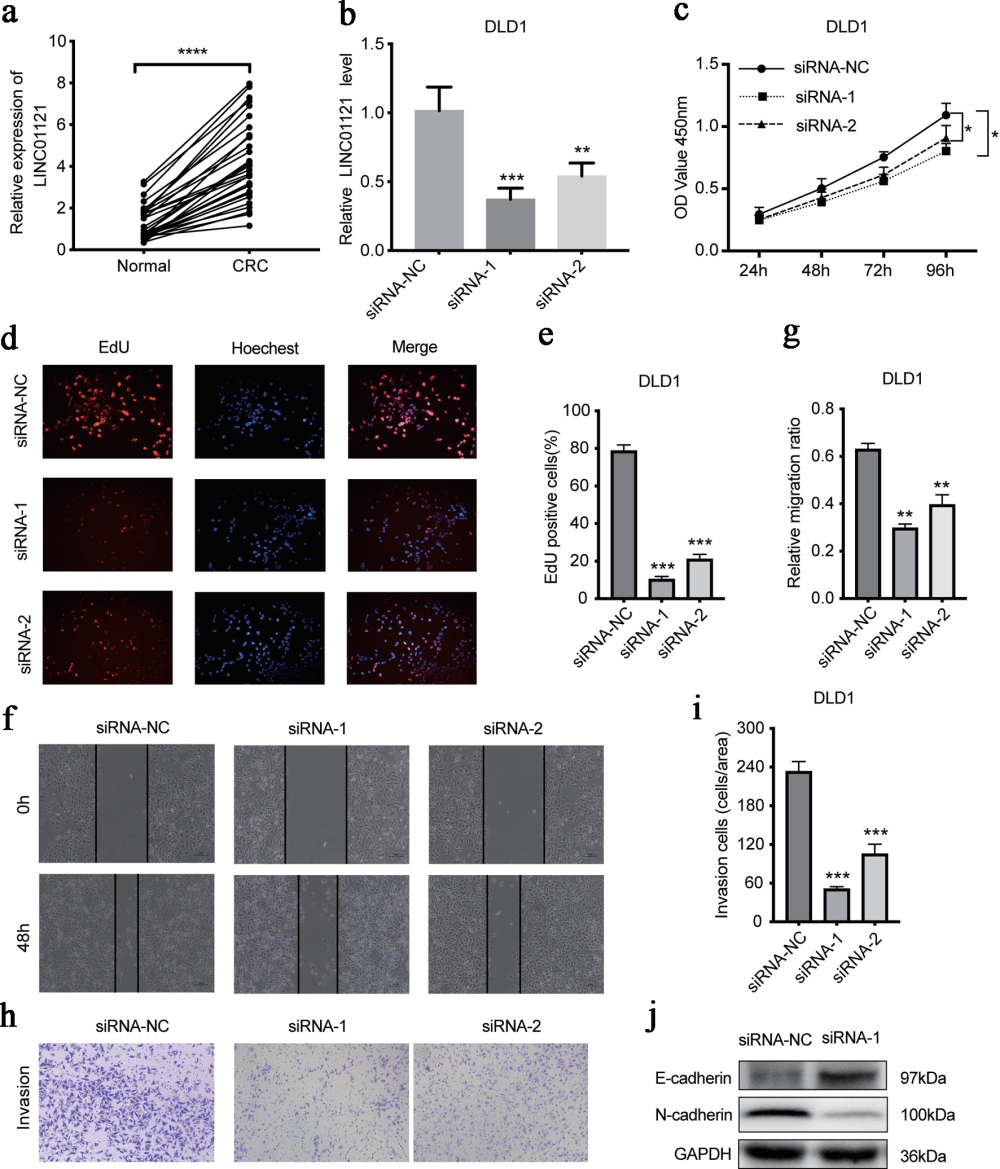
LINC01121 mediates the proliferation, migration, and invasion of DLD1 cells. (a) The expression level of LINC01121 in CRC tissue and adjacent tissues. (b) RT-qPCR was used to access the expression level of LINC01121 in DLD1 after knockdown. (c-e) CCK8 and EdU assays assessed the cell proliferation levels after LINC01121 knockdown. (f, g) Scratch assays were performed to detect the cell migration ability after LINC01121 knockdown. (h, i) Transwell invasion assays showed the cell invasion ability after LINC01121 knockdown. (j) Western blotting revealed the change of E-cadherin and N-cadherin expression after LINC01121 knockdown. Data are represented as mean ± SD. P values were calculated via one-way ANOVA test. **P < 0.01, ***P < 0.001, ****P < 0.0001. ANOVA: analysis of variance; CRC: colorectal cancer; RT-qPCR: reverse transcription-quantitative polymerase chain reaction.

To verify the functional role of LINC01121 in CRC, we first detected the LINC01121 level in CRC tissues. In paired specimens, its level in CRC was notably greater than in the adjacent samples (P < 0.0001) (Fig. 6a). Subsequently, we transfected DLD1 cell line with LINC01121 siRNA and detected the knockdown efficiency by reverse transcription-quantitative polymerase chain reaction (RT-qPCR) (Fig. 6b). The CCK8 experiments showed that knocking down LINC01121 significantly reduced DLD1 cells proliferation (Fig. 6c). EdU assays also showed a significant decrease in cell proliferation after LINC01121 knockdown (Fig. 6d, e). The scratch experiments indicated that the LINC01121downregulation significantly inhibited cell migration ability (Fig. 6f, g). In the transwell experiments, it was observed that the downregulation of LINC01121 remarkably suppressed the invasive capacity (Fig. 6h, i). Western blotting revealed that E-cadherin expression increased while N-cadherin decreased after LINC01121 knockdown (Fig. 6j).

## Discussion

Despite significant research efforts over the years to improve our understanding of the development and treatment modalities of CRC, the prognosis for patients remains unsatisfactory. Therefore, it is necessary to identify relevant biomarkers, elucidate the underlying molecular mechanisms of CRC, and develop effective interventions to improve patient outcomes.

LncRNA is widely distributed and has numerous functions in cells. LncRNAs participate in epigenetic gene regulation, forming scaffolds to organize DNA regions and regulate transcription, recruiting RNA and cytoplasmic factors to post transcriptional control sites, and serving as assembly platforms for multi-protein complex functional connections, in fact, they achieve a hypergenome layer for protein expression programs and cell fate [13]. In CRC, lncRNAs also play significant roles. For example, recent studies have shown that LncRNA-HMG can protect CRC cells from ferroptosis during chemotherapy, thereby enhancing their drug resistance [14]. LINC00982 may express a novel protein, PRDM16-DT, which functions as a new regulator in CRC metastasis and drug resistance [15]. Additionally, LOC101928222, through the m6A-dependent pathway, cooperates with IGF2BP1 to stabilize HMGCS2 mRNA, thus promoting cholesterol synthesis and ultimately driving CRC progression [16]. In our current study, we focused on LINC01121, given our limited understanding of its role in cancer. To comprehensively analyze its biological functions in CRC and uncover its associated regulatory pathways, we combined data from publicly available databases with *in vitro* experimental validation.

Previous research has indicated that the downregulation of LINC01121 significantly inhibits the development of breast cancer cells. Furthermore, LINC01121 directly interacts with miR-150-5P, leading to the increase of HMGA2 level, which regulates metastasis in breast cancer [9]. Additionally, pterostilbene has been found to inhibit both the proliferation and EMT of breast cancer cells by decreasing LINC01121 expression [10]. Moreover, LINC01121 is implicated in facilitating the metastasis of prostate cancer cells through the activation of the EMT process [17].

In our research, we found that LINC01121 was significantly overexpressed in CRC, and its overexpression was related to worse prognosis. Functional enrichment analysis revealed that LINC01121 was associated with cell proliferation, NRAS signaling pathway, EMT, CRC MYC, primary immunodeficiency syndrome, and G1S phase transition of mitosis.

Tumor-infiltrating immune cells play a crucial role in tumorigenesis, development, and therapeutic efficacy [18, 19]. Thus, understanding the types of infiltrating immune cells may provide insights into the mechanisms by which LINC01121 is involved in CRC. We found that LINC01121 was negatively correlated with anti-tumor immune cells, such as NK CD56bright cells, Th17 cells, and Cytotoxic cells. NK CD56 bright cells are a subset of natural killer (NK) cells characterized by high CD56 level and low level of CD16 expression, possessing powerful cytotoxic activity and immune regulatory functions [20]. In tumors, NK CD56bright cells play various important roles. On one hand, they have direct cytotoxic effects on tumor cells. These cells can recognize and eliminate cancer cells by releasing cytotoxic granules and promoting apoptosis, thereby inhibiting tumor growth and spread. On the other hand, NK CD56bright cells also participate in immune regulation. They can secrete various cytokines and chemokines, such as interferon-gamma (IFN-γ), tumor necrosis factor-alpha (TNF-α), CCL3, and CCL4, which can activate other immune cells, such as macrophages and T cells, enhancing the overall anti-tumor immune response. Additionally, NK CD56 bright cells are closely related to the effectiveness of anti-tumor immunotherapy. They can play a significant role in checkpoint inhibitor therapy by enhancing T-cell responses and modulating the tumor microenvironment to improve therapeutic efficacy [21, 22]. Th17 cells are a specific subset of T cells characterized by the production of interleukin (IL)-17 and other inflammatory factors [23]. They can promote the infiltration and activation of tumor-associated immune cells, such as CD8^+^ T cells, NK cells, and macrophages, thereby enhancing the immune killing effects against tumor cells. Furthermore, Th17 cells can guide immune responses by stimulating the function of dendritic cells, improving anti-tumor immunity. However, Th17 cells may also mediate inflammatory responses that lead to congestion and tissue damage, potentially stimulating tumor growth and invasion [24, 25]. Cytotoxic cells, primarily including CD8^+^ T cells and NK cells, play crucial roles in tumor immunity, mainly through directly killing tumor cells or releasing cytokines to regulate immune responses [26, 27].

In addition, the LINC01121 level is negatively correlated with the infiltration of eosinophils. In recent years, studies have found that eosinophils also play important roles in the tumor microenvironment, with effects that can either promote tumor development or exert anti-tumor activity, depending on the tumor type and microenvironment [28]. Eosinophils can directly kill tumor cells by releasing cytotoxic granules, such as major basic protein and eosinophil cationic protein. They are also capable of secreting various cytokines, including IFN-γ, TNF-α, and IL-4, which can activate other immune cells and enhance anti-tumor immune responses. The quantity of eosinophils in tumors may serve as a prognostic marker in certain immunotherapies. An increase in eosinophils could be associated with patient responses to immune checkpoint inhibitors [29, 30].

To better understand the effect of LINC01121 in CRC, *in vitro* experiments were performed. The results demonstrated that LINC01121 promoted the proliferation, migration, and invasion ability of CRC cells. However, there are still limitations, thus *in vitro* experiments and future investigations in mechanism are needed to further explore the functions of LINC01121 in CRC.

### Conclusion

In this study, we report for the first time that elevated expression of LINC01121 is notably correlated with disease progression, poor survival, and immune infiltration in CRC. High levels of LINC01121 in CRC facilitate cell proliferation, migration, and invasion. Furthermore, LINC01121 may serve as a potential predictor of treatment outcomes and could emerge as a new biomarker for CRC. This research offers valuable insights into the clinicopathological significance and molecular mechanisms underlying CRC.

## Data Availability

The data used to support the findings of this study are included within the article. The data and materials in the current study are available from the corresponding author on reasonable request.
